# Midnight Musings: Exploring Sleep, Home Environment, and Mental Health in Hispanic/Latino Communities

**DOI:** 10.1093/geroni/igaf122.2490

**Published:** 2025-12-31

**Authors:** Aziza Siddiqui, Srujana Chekuri, Julie Blaskewicz Boron, Ibrahim Bilau, Eunhwa Yang

**Affiliations:** University of Nebraska Omaha, Omaha, Nebraska, United States; University of Nebraska Omaha, Elkhorn, Nebraska, United States; University of Nebraska Omaha, Omaha, Nebraska, United States; Georgia Institute of Technology, Atlanta, Georgia, United States; Georgia Institute of Technology, Atlanta, Georgia, United States

## Abstract

Sleep quality is closely tied to home environment factors including safety and satisfaction with indoor environmental conditions, which significantly influence mental health outcomes. In Hispanic/Latino communities, where resource access may be limited, the home environment may disproportionately impact well-being, particularly for older adults. This study examined the interplay between sleep patterns, home satisfaction, safety, and mental health in aging Hispanic/Latino adults (N = 30, mean age=67.1, SD = 5.24, range= 60-82). A series of regression analyses were used to examine the relationship between home environmental factors and self-reported mental health outcomes. The results indicated that home safety (p < 0.05) and concerns about sleep problems (p < 0.05) were significantly associated with self-reported depression scores. Similarly, impaired quality of life (p < 0.05) and concerns about sleep problems (p < 0.05) were significantly associated with self-reported anxiety scores. In line with these findings, significant correlations emerged between poor sleep quality and higher levels of depression (r=.49, p=.003) and anxiety (r=.34, p=.03). Lower home satisfaction was associated with higher depression (r=-.39, p=.01) and anxiety (r=-.63, p<.001). These findings highlight the urgent need for innovative approaches to aging in underrepresented communities, emphasizing the critical role of the home environment in improving sleep, mental health, and overall well-being. This research underscores the need for culturally tailored innovations in housing and mental health support to improve outcomes for underserved aging populations. Future initiatives should integrate physical (e.g., adaptive home design) and emotional (e.g., social support) elements to enhance the overall aging experience.

